# Free-Radical Scavenging Properties and Reducing Power of Grape Cane Extracts from 11 Selected Grape Cultivars Widely Grown in China

**DOI:** 10.3390/molecules161210104

**Published:** 2011-12-06

**Authors:** Ang Zhang, Yulin Fang, Hua Wang, Hua Li, Zhenwen Zhang

**Affiliations:** 1 College of Enology, Northwest A&F University, Yangling, Shaanxi 712100, China; 2 Shaanxi Engineering Research Center for Viti-Viniculture, Yangling, Shaanxi 712100, China

**Keywords:** grape cane extracts, antioxidant activity, antiradical effect, phenolic compounds

## Abstract

Total phenolic contents (TPC), total flavonoid contents (TFC), related antioxidative and antiradical capabilities of grape cane extracts from 11 varieties (five *V. vinifera* cultivars and six Chinese wild grapes) widely grown in China were evaluated. Antioxidant properties were determined as DPPH and ABTS radical-scavenging abilities, superoxide anion and hydroxyl radical and hydrogen peroxide scavenging assays, as well as reducing power. Phenolic profiles of the extracts were characterized by using high-performance liquid chromatography (HPLC) techniques. All extracts exhibited strong antioxidant and effective free radical inhibition activities (EC_50_ values), which generally correlated negatively with TPC (*r* = −0.804 to −0.918) and TFC (r = −0.749 to −0.888). In comparison with gallic acid, Trolox and *tert*-butylhydroquinone (positive controls), most grape cane extracts showed more efficient scavenging effects toward different reactive oxygen species. HPLC analysis revealed the presence of (+)-catechin, (−)-epicatechin, and *trans*-resveratrol as major phenolic components in the extracts. These results suggest that grape cane extracts may serve as a potential source of natural antioxidant for food and pharmaceutical application.

## 1. Introduction

Reactive oxygen species (ROS), the by-products of cell metabolism, include free radicals such as superoxide anion radical and hydroxyl radical and non-free-radicals such as hydrogen peroxide and singlet oxygen [1]. ROS are continuously produced during normal physiologic activities and may not have harmful effects to cell function at physiological concentrations. Harmonious cellular metabolism systems are characterized by the perfect balance between oxidant challenge and antioxidant response. ROS are also excessively generated in living organisms when exposed to ultraviolet rays, ozone, tobacco smoke, industrial exhausts and other exogenous stress factors [2]. They can stimulate free radical chain reactions subsequently damaging the cellular bio-molecules such as nucleic acids, lipids and proteins [2]. When ROS formation exceeds the capacity of cellular antioxidant defenses to neutralize their effects, the delicate cellular balance will be disturbed; furthermore, various pathophysiological disorders such as arthritis, diabetes, inflammation, cancer and aging process can ensue [3,4]. On the other hand, lipid oxidation initiated by free radicals is believed to be a major cause of food deterioration, affecting color, flavor, texture and nutritional value [5].

Antioxidants can terminate or retard the oxidation process by scavenging free radicals. Recent epidemiological studies have revealed the associations between the consumption of antioxidant-rich foods and the prevention of oxidative-stress-related diseases [6,7,8]. However, synthetic antioxidants such as butylated hydroxyanisole (BHA), butylated hydroxytoluene (BHT) and *tert*-butylhydroquinone (TBHQ) are restricted by legislative rules because of doubts over their toxicity and carcinogenicity in many countries [9,10]. In order to protect foods and human beings against oxidative damage, considerable attention has been paid to explore the natural and safer antioxidants, which could be used for human consumption. Among the dietary antioxidants, phenolic compounds, secondary metabolites occurring in plants, are the most abundant natural antioxidants [11].

Grape canes, the main solid wastes from vineyards [12], have been shown to be a rich source of high-added-value bioactive compounds, including phenolic acids, flavonoids and stilbenes [12,13,14,15]. These compounds possess strong antioxidant properties that enable them to scavenge free radicals, donate hydrogen, chelate metals, break radical chain reactions, and quench singlet oxygen *in vitro* and *in vivo* [6,16,17]. Grapevines are pruned annually, after which the prunings are disposed of in landfills, burned *in situ* or as fuels, indicating low-value utilizations [18]. China, as one of the most important centers of origin of *Vitis*, has achieved much success in development of grape and wine industry in the past two decades. Based on a conservative estimate, annual yield of fresh grape cane wastes in China is more than two million tons [19]. In recent years, researchers are making great efforts in investigating the antioxidant capacities of grape pomace, seed and stems while neglecting grape canes. As a part of our ongoing work on potential utilization of grape cane wastes, antioxidant properties of crude methanolic extracts from grape canes of five widely-grown *V. vinifera* cultivars and six main Chinese wild grapes were assessed using different methods *in vitro* such as free radical-scavenging and reducing power capabilities. In parallel, the phytochemical contents and the main phenolic constituents of the methanolic extracts were characterized and quantified by colorimetric methods and HPLC-DAD-UV techniques.

## 2. Results and Discussion

### 2.1. Extraction Yields (EY), Total Phenolic Content (TPC) and Total Flavonoid Content (TFC)

**Figure 1:**
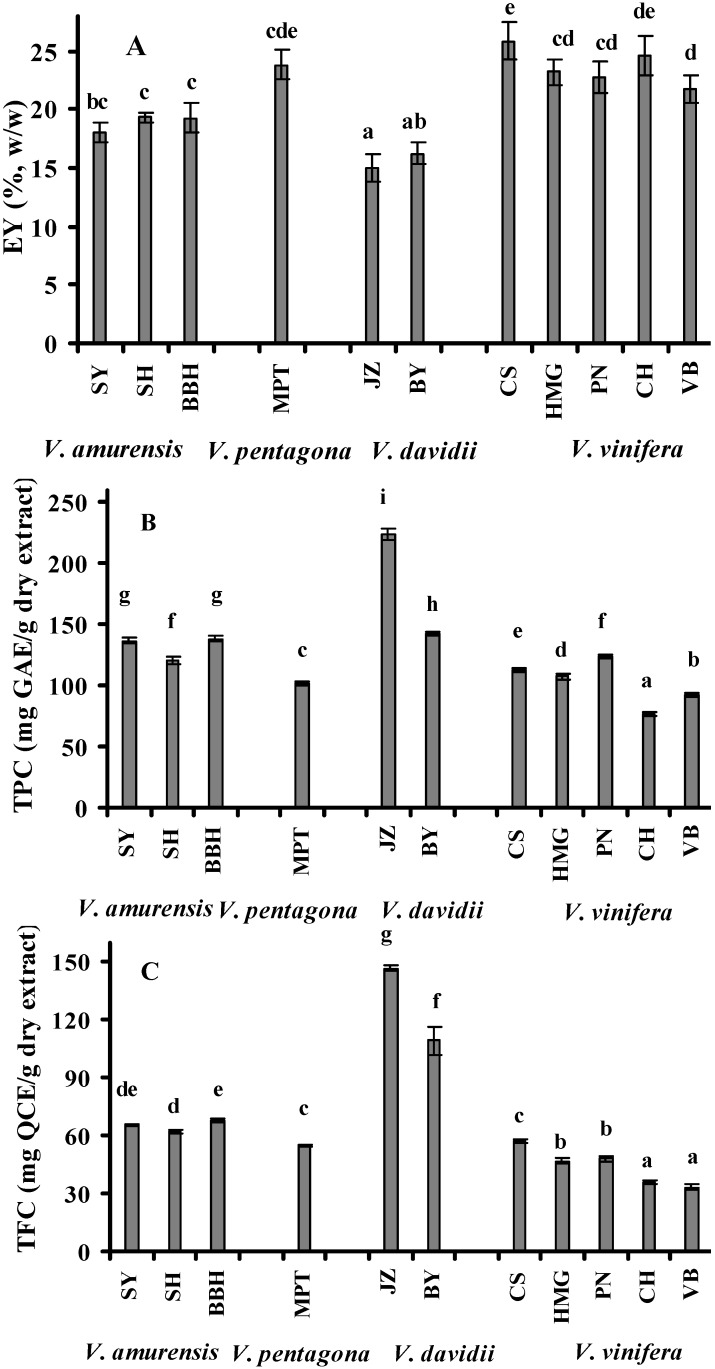
Comparative analysis of extraction yields (EY, g/100 g, w/w) (**A**), total phenolic contents (TPC, mg GAE/g dry extract) (**B**) and total flavonoid contents (TFC, mg QCE/g dry extract) (**C**) of the grape cane extracts from different cultivars. SY: Shuangyou; SH: Shuanghong; BBH: Beibinghong; MPT: Maoputao; JZ: Junzi; BY: Baiyu; CS: Cabernet Sauvignon; HMG: Hongmeigui; PN: Pinot Noir; CH: Chardonnay; VB: Victoria Blanc. Experiments were triplcated and values are presented as means ± SD. Bars with different letters denote significant differences at *p* < 0.05.

Methanol is one of the most widely used solvents for extracting polyphenols from solid grape wastes [12,20,21]. Aqueous methanol was found to be more effective at extracting polyphenols linked to polar fibrous matrices [22]. The main constituents of grape canes such as lignin, cellulose and hemicelluloses can be hydrolyzed on the presence of strong acid in the extraction medium [23]. With these facts in mind, in this study, acidified methanol was used as our extraction medium and the results of grape cane EY are presented in Figure 1A. The average EY of different grape species were in a descending order: *V. pentagona* (23.9%) ≥ *V. vinifera* (23.6%) > *V. amurensis* (18.9%) > *V. davidii* (15.6%). The EY values of grape cultivars ‘Junzi’ (14.9%) and ‘Baiyu’ (16.3%) from *V. davidii* were significantly lower than those from other species (*p* < 0.05). The much higher yields of grape canes from ‘Cabernet Sauvignon’, ‘Chardonnay’ and ‘Maoputao’ might be attributed to their higher contents of the soluble components compared to other cultivars.

It has been recognized that phenolic compound contents of botanical materials are associated with their antioxidant activities [24]. Flavonoids found ubiquitously in plants are the most common group of phytophenolics [25]. The TPC and TFC of all grape cane extracts varying from 76.4 to 224.5 mg gallic acid equivalent (GAE)/g extract and 33.1 to 146.6 mg quercetin equivalent (QCE)/g extract were compared in Figures 1B and 1C, respectively. Considerable variability in the values of TPC and TFC among four grape species was observed. Within the same grape species, the TPC and TFC of red varieties were significantly higher than those of white ones (*p* < 0.05). The TPC of the investigated grape species were in the following order: *V. davidii* > *V. amurensis* > *V. vinifera* > *V. pentagona*, with the mean TPC of 183.4, 132.0, 102.5 and 101.9 mg GAE/g extract, respectively. The grape cultivar ‘Junzi’ (224.5 mg GAE/g extract) had the highest TPC and ‘Baiyu’ (142.3 mg GAE/g extract) came next, whereas ‘Chardonnay’ (76.4 mg GAE/g extract) presented the lowest contents followed by ‘Victoria Blanc’ (92.2 mg GAE/g extract). The order of the TFC for the grape species analyzed was as follows: *V. davidii* > *V. amurensis* > *V. pentagona* > *V. vinifera* with the average TFC of 128.1, 65.3, 54.9 and 44.3 mg QCE/g extract, respectively. The TFC of ‘Junzi’ (146.6 mg QCE/g extract) from *V. davidii* was approximately 2–5 folds higher than those of from other species. Various factors such as variety, growing conditions and viticultural managements might be responsible for the observed differences in phytochemicals contents [26,27]. In present study, the levels of total phenolic and flavonoid contents for 11 grape cultivars analyzed ranged from 18.8 to 33.6 GAE/g cane powder and 4.4 to 7.5 mg QCE/g cane powder, respectively, which were calculated based on the extraction yields.

The significant correlation (*r* = 0.930, *p* < 0.01) between the TPC and TFC from correlation analysis suggested that flavonoids are a major compounds contributing to total phenolics in grape cane extracts (Table 1). The grape cane extracts with high EY values had low levels of TPC and TFC, indicating that quantitative efficiency of extraction were not directly related to their qualitative efficiency. Similar behaviors were also found in grape pomace extracts (*V. vinifera*) [28] and buckwheat extracts [29]. As far as we know there is only one published report on the total phenolic content of grape cane. In this report the TPC of ‘Pinot Noir’ (*V. vinifera*) was expressed as resveratrol equivalents (8.9 mg resveratrol/g dry ethanolic extract) [15].

**Table 1:** Pearson’s correlation coefficients (*r*) of EC_50_ values of antioxidant activities, total phenolic contents (TPC) and total flavonoid contents (TFC) in grape cane extracts (*n* = 11).

	TPC	TFC	DPPH^•^	ABTS^•^^+^	O_2_^•−^	OH^•^	H_2_O_2_	Reducing Power
TPC	1	0.930 **	−0.866 **	*−*0.893 **	*−*0.896 **	*−*0.885 **	*−*0.918 **	*−*0.804 **
TFC		1	*−*0.855 **	*−*0.888 **	*−*0.766 **	*−*0.754 **	*−*0.872 **	*−*0.749 **

** Correlation is significant at the 0.01 level (two-tailed).

### 2.2. Antioxidant Activities of Grape Cane Extracts

Radical scavenging activity is very important due to the deleterious role of free radicals in foods and biological systems. In the present study, the antioxidant properties of grape cane extracts were evaluated by several widely used assays: DPPH and ABTS radical-scavenging assays, superoxide anion, hydroxyl radical and hydrogen peroxide scavenging assays and reducing power assay.

#### 2.2.1. DPPH Radical-Scavenging Activity

**Figure 2:**
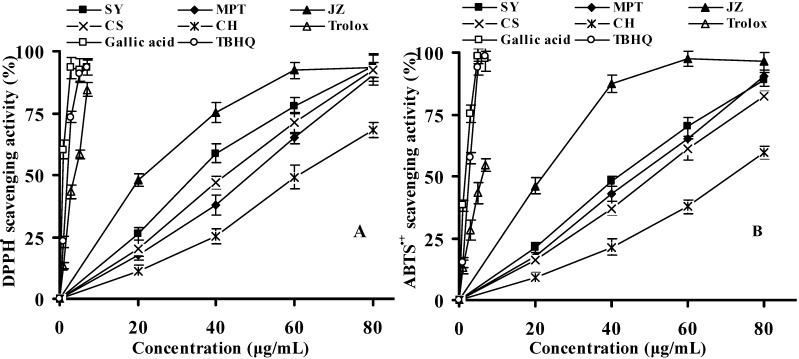
DPPH (**A**) and ABTS (**B**) radical-scavenging activities of grape cane extracts and positive controls. SY: Shuangyou; MPT: Maoputao; JZ: Junzi; CS: Cabernet Sauvignon; CH: Chardonnay. Experiments were triplicated and values are presented as means ± SD.

DPPH radical (DPPH^•^) scavenging effects of 11 grape cane extracts (at the concentrations of 20, 40, 60, and 80 μg/mL) and standard antioxidants (at the concentrations of 1, 3, 5, and 7 μg/mL) were investigated. The representative grape cultivars (‘Shuangyou’ of *V. amurensis*, ‘Maoputao’ of *V. pentagona*, ‘Junzi’ of *V. davidii*, ‘Cabernet Sauvignon’ and ‘Chardonnay’ of *V. vinifera*) were selected to plot a clear graph for comparison (Figure 2A). Grape cane extracts and positive controls clearly demonstrated a dose-dependent antioxidant activity against DPPH^•^. At a concentration of 20 μg/mL, the cultivar ‘Junzi’ obtained the highest percent scavenging activity (48.2 ± 2.5%) among all the extracts (*p* < 0.05), while ‘Chardonnay’ yielded the lowest (11.3 ± 2.3%). At the concentration of 80 μg/mL, all the extracts in Figure 2A exhibited a similar scavenging effect around 93% except ‘Chardonnay’ (68.3 ± 2.9%), which might be indicative of maximal inhibition. Gallic acid, Trolox and TBHQ displayed higher radical-scavenging effects than did all extracts at the same concentration range (1–7 μg/mL). 

With regard to EC_50_, as shown in Table 2, amongst all extracts examined, the cultivar ‘Junzi’, with the lowest EC_50_ (21.97 ± 0.93 μg/mL), indicated the highest DPPH^•^ scavenging activity, followed by ‘Baiyu’ (30.70 ± 0.89 μg/mL), whilst ‘Chardonnay’ with the highest EC_50_ value (60.88 ± 1.53 μg/mL) exhibited the lowest scavenging activity. Overall, the DPPH^•^ scavenging activity was found to be in the order of: Gallic acid > TBHQ > Trolox > Junzi > Baiyu > Shuangyou > Victoria Blanc > Beibinghong > Cabernet Sauvignon > Pinot Noir > Maoputao ≥ Shuanghong > Hongmeigui > Chardonnay. A correlation analysis was carried out between phenolic and flavonoid contents and EC_50_ values of DPPH^•^ scavenging assay for all cultivars (Table 1). The EC_50_ values negatively and significantly associated the TPC (*r* = −0.866, *p* < 0.01) and TFC (*r* = −0.855, *p* < 0.01), indicating a positive relationship between the DPPH^•^ scavenging activities and the total phenolic contents. The results suggested that the phenolic compounds contributed significantly to the antioxidant capacities of grape cane extracts. Similar relationships have been widely reported in many plants [24,29]. Although there is no information available in the literatures on the DPPH^•^ scavenging activity of grape cane extract, current EC_50_ values of gallic acid and Trolox are in a good agreement with those in the same experimental conditions reported by Villaño *et al*. [30].

#### 2.2.2. ABTS Radical-Scavenging Activity

ABTS assay is more versatile as both the polar and non-polar samples can be evaluated for their scavenging activity. The spectral interference is minimized since the absorption maximum used is around 734 nm (a wavelength not normally encountered by natural products) [31].

ABTS radical (ABTS^•+^) scavenging abilities of grape cane extracts (20, 40, 60, and 80 μg/mL) were compared with standard antioxidants (1, 3, 5, and 7 μg/mL) (Figure 2B). Gallic acid, Trolox and TBHQ, as expected, were much more effective than grape cane extracts at all concentrations tested. The dose-dependent scavenging of all cultivars on ABTS^•+^ in Figure 2B showed a similar trend with the results of the DPPH^•^ assay except ‘Cabernet Sauvignon’ and ‘Maoputao’, which showed an opposite tendency in these two assays. These differences could be due to the different stoichiometry reactions between the grape cane extracts and the DPPH^•^ and ABTS^•+^. In addition, the compositional differences in extracts and their solubility in different testing systems may also affect their capacities to act as antioxidants [32,33].

It can be seen from Table 2 that the hierarchy of ABTS^•+^ scavenging effects based on the EC_50_ values was gallic acid > TBHQ > Trolox > Junzi > Baiyu > Shuangyou > Beibinghong > Maoputao ≥ Pinot Noir > Victoria Blanc > Shuanghong > Cabernet Suavignon > Hongmeigui > Chardonnay. The EC_50_ values of grape cane extracts negatively and strongly correlated with TPC (*r* = −0.893, *p* < 0.01) and TFC (*r* = −0.888, *p* < 0.01), suggesting their great contribution in scavenging ABTS^•+^ in the present work. Karacabey and Mazza used the Trolox equivalent antioxidant capacity (TEAC) assay to evaluate the antioxidant activity of ethanolic extract of grape cane (cv., Pinot Noir of *V. vinifera*) and reported a predicted TEAC value of 260.8 μM Trolox equivalents/g dry grape cane [15]. Different ways of expressing the antioxidant capacity made it difficult to compare the results from the similar samples, even for the same method.

**Table 2:** EC_50_^a^ values of grape cane extracts in DPPH radicals (DPPH^•^), ABTS radicals (ABTS^•+^), hydrogen peroxide (H_2_O_2_), superoxide radicals (O_2_^•^^－^) and hydroxyl radicals (OH^•^) scavenging and reducing power (RP) assays.

Species/cultivars	DPPH^•^ EC_50_	ABTS^•+^ EC_50_	H_2_O_2_ EC_50_	O_2_^•−^ EC_50_	OH^•^ EC_50_	RP EC_50_
	(μg/mL) ^b^	(μg/mL) ^b^	(mg/mL) ^b^	(mg/mL) ^b^	(mg/mL) ^b^	(μg/mL) ^c^
***V. amurensis***						
Shuangyou	36.40 ± 1.03 e	42.73 ± 0.55 e	0.07 ± 0.00 b	0.10 ± 0.00 b	0.31 ± 0.02 b	62.14 ± 2.60 d
Shuanghong	49.80 ± 0.93 i	52.47 ± 2.55 hi	0.10 ± 0.01 cde	0.13 ± 0.01 cde	0.40 ± 0.02 d	70.32 ± 3.65 fg
Beibinghong	43.09 ± 1.45 fg	45.76 ± 0.80 f	0.09 ± 0.00 bcd	0.12 ± 0.00 bc	0.36 ± 0.02 c	64.27 ± 3.25 de
***V. pentagona***						
Maoputao	49.06 ± 1.22 i	50.06 ± 1.13 g	0.12 ± 0.01 efg	0.16 ± 0.00 fg	0.48 ± 0.02 f	82.44 ± 1.75 h
***V. davidii***						
Junzi	21.97 ± 0.93 c	23.64 ± 0.58 c	0.04 ± 0.00 a	0.07 ± 0.00 a	0.21 ± 0.01 a	33.24 ± 2.26 c
Baiyu ^w^	30.70 ± 0.89 d	33.70 ± 1.11 d	0.08 ± 0.00 bc	0.12 ± 0.00 cd	0.39 ± 0.01 cd	65.32 ± 4.07 def
***V. vinifera***						
Cabernet Sauvignon	44.00 ± 1.43 gh	54.00 ± 1.43 i	0.10 ± 0.01 def	0.15 ± 0.01 ef	0.48 ± 0.01 f	65.83 ± 2.43 def
Hongmeigui	52.30 ± 1.39 j	60.97 ± 1.25 j	0.10 ± 0.01 def	0.12 ± 0.01 cd	0.43 ± 0.02 e	62.28 ± 1.71 d
Pinot Noir	45.51 ± 1.11 h	50.17 ± 1.91 g	0.12 ± 0.01 efg	0.14 ± 0.00 def	0.44 ± 0.01 e	69.25 ± 2.01 efg
Chardonnay ^w^	60.88 ± 1.53 k	71.55 ± 1.80 k	0.15 ± 0.01 h	0.17 ± 0.01 g	0.59 ± 0.01 g	138.8 ± 4.13 j
Victoria Blanc ^w^	42.00 ± 1.38 f	51.67 ± 1.64 gh	0.12 ± 0.01 fg	0.13 ± 0.01 cde	0.40 ± 0.03 d	95.94 ± 2.15 i
***Positive controls***						
Gallic acid	0.85 ± 0.03 a	1.35 ± 0.09 a	0.25 ± 0.02 i	0.52 ± 0.02 h	1.48 ± 0.02 h	21.36 ± 3.93 a
Trolox	3.86 ± 0.04 b	6.50 ± 0.56 b	0.33 ± 0.03 j	0.79 ± 0.02 j	2.79 ± 0.02 i	73.25 ± 2.65 g
TBHQ	1.90 ± 0.07 a	2.53 ± 0.18 a	0.13 ± 0.01 gh	0.62 ± 0.02 i	0.59 ± 0.01 g	26.81 ± 1.92 b

^a^ Values (mean ± SD, *n* = 3) with the same lowercases are not significantly different within each column according to Duncan's new multiple range test (*p* < 0.05). ^b^ EC_50_: effective concentration at which 50% radicals are scavenged. ^c^ EC_50_: effective concentration at which the absorbance is 0.5. ^w^ White grape cultivars. Others are red ones.

#### 2.2.3. Reactive Oxygen Species (ROS) Scavenging Activity

**Figure 3:**
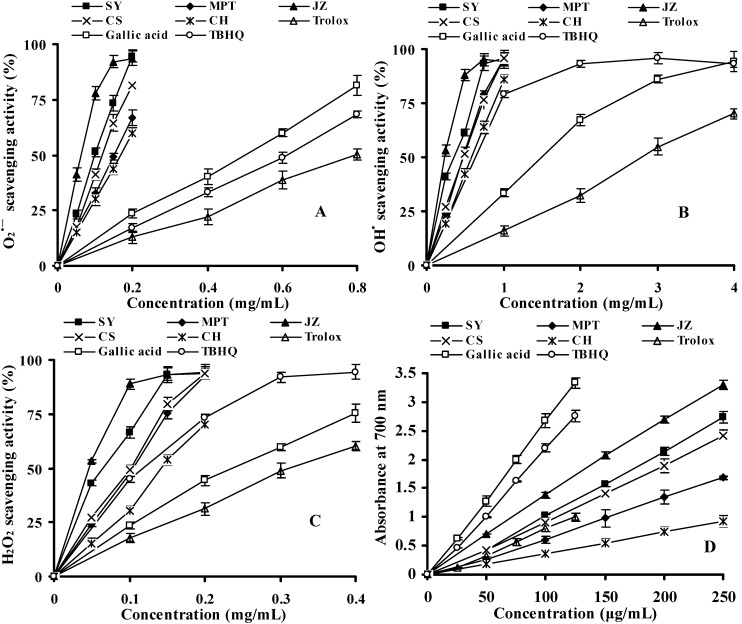
Superoxide radicals (**A**), hydroxyl radicals (**B**) and hydrogen peroxide (**C**) scavenging activities and reducing power (D) of grape cane extracts and positive controls. SY: Shuangyou; MPT: Maoputao; JZ: Junzi; CS: Cabernet Sauvignon; CH: Chardonnay. Experiments were triplicated and values are presented as means ± SD.

Unlike DPPH and ABTS radicals, which are less relevant to the biological systems, ROS are commonly found in living tissues. In spectrophotometric measurements, superoxide anions (O_2_^•−^) derived from the PMS/NADH/O_2_ system reduces the yellow colored NBT to form the blue formazan [34], which can be inhibited by the addition of antioxidants to reaction mixture. Grape cane extracts (0.05, 0.1, 0.15 and 0.2 mg/mL) and standard antioxidants (0.2, 0.4, 0.6 and 0.8 mg/mL) shown in Figure 3A exhibited dose-dependent O_2_^•−^ scavenging activities. Among all the cultivars, ‘Shuangyou’ had the highest scavenging activity (93.5 ± 2.6%) at 0.2 mg/mL, while ‘Chardonnay’ showed the lowest activity (59.9 ± 2.3%). However, the value was much higher than that of gallic acid (23.5 ± 2.1%). With regard to the EC_50_ values, all the investigated extracts generally exerted the significantly higher superoxide radical-scavenging capacities than did the positive standards (*p* < 0.05), such as Trolox and gallic acid, which possessed comparable EC_50_ values with those documented in previous studies [35,36]. Superoxide radical-scavenging activities based on the EC_50_ values were in the following order: Junzi > Shuangyou > Beibinghong > Hongmeigui ≥ Baiyu > Shuanghong ≥ Victoria Blanc > Pinot Noir ≥ Cabernet Sauvignon > Maoputao > Chardonnay > Gallic acid > TBHQ > Trolox (Table 2). The correlation analysis showed that EC_50_ values of grape cane extracts were negatively and strongly associated with TPC (*r* = −0.896, *p* < 0.01) and TFC (*r* = −0.766, *p* < 0.01) (Table 1), indicating the phenolic and flavonoids present in the extracts for governing the O_2_^•−^ scavenging activities observed.

Hydroxyl radical (OH^•^) has the potential of reacting with almost every cellular macromolecules and thereby inducing tissue damage [37,38]. In the present assay, OH^•^ were derived from the FeSO_4_-EDTA/H_2_O_2_ system via Fenton reaction [39]. Grape cane extracts (0.25, 0.5, 0.75 and 1 mg/mL) and positive controls (1, 2, 3 and 4 mg/mL) exhibited concentration-dependent OH^•^ scavenging activities. When at 1 mg/mL, the scavenging activities of the extracts in Figure 3B ranged from 85.9 ± 2.3% for ‘Chardonnay’ to 96.0 ± 1.6% for ‘Cabernet Sauvignon’. Meanwhile, standard gallic acid, Trolox and TBHQ scavenged OH^•^ by 33.5 ± 1.8%, 15.9 ± 2.2% and 78.9 ± 1.7% at the same concentration, respectively. The result suggested that grape cane extracts were the primary hydroxyl radical scavengers. Overall, the OH^•^ scavenging activities based on EC_50_ values were ranked in the order: Junzi > Shuangyou > Beibinghong > Baiyu > Shuangyou ≥ Victoria Blanc > Hongmeigui ≥ Pinot Noir > Maoputao ≥ Cabernet Sauvignon > TBHQ > gallic acid > Trolox. Comparing the phenolic and flavonoid contents with the EC_50_ values of all extracts, the correlation coefficients (Table 1) were negatively high (*r* = −0.885 and −0.754 for TPC and TFC, respectively, *p* < 0.01). These results indicated that strong OH^•^ scavenging activities of grape cane extracts were closely related to their high levels of phenolic compounds and due to the scavenging of the radical by hydrogen donation. Makris *et al*. also showed a positive association between TPC and OH^•^ scavenging activities of winery waste extracts [21].

Hydrogen peroxide (H_2_O_2_) is a non-radical ROS in living organisms and has the ability to penetrate cell membranes, inactivate enzymes by oxidation of thiol groups, and initiate lipid peroxidation. The scavenging capacities of H_2_O_2_ by grape cane extracts (0.05, 0.1, 0.15 and 0.2 mg/mL) along with standard antioxidants (0.1, 0.2, 0.3 and 0.4 mg/mL) are shown in Figure 3C. All the extracts were capable of scavenging H_2_O_2_ in a dose-dependent manner. At a concentration of 0.2 mg/mL, all grape cane extracts in Figure 3C exhibited significantly higher , (*p* < 0.05) scavenging abilities (92.9–94.5%) than did positive controls (31.2 ± 2.7%, 44.3 ± 2.5% and 73.2 ± 2.3% for Trolox, gallic acid and TBHQ, respectively) except the cultivar ‘Chardonnay’ (69.9 ± 2.0%). As shown in Table 2, hydrogen peroxide scavenging activities based on the EC_50_ values were in the following order: Junzi > Shuangyou > Baiyu > Beibinghong > Shuanghong > Cabernet Sauvignon ≥ Hongmeigui > Pinot Noir ≥ Maoputao > Victoria Blanc > TBHQ > Chardonnay > Gallic acid > Trolox. In the present analysis it was found that the EC_50_ values of grape canes extracts were negatively and strongly associated with TPC (*r* = −0.918, *p* < 0.01) and TFC (*r* = −0.872, *p* < 0.01) (Table 1). These results suggested that phenolic compounds in grape canes with electron-donating capacities may govern the H_2_O_2_ scavenging activities of the tested extracts. Although H_2_O_2_ is not a highly reactive molecule, it can sometimes be toxic to cells and food systems because it may give rise to hydroxyl radicals and singlet oxygen by reacting with transition metal ions [5]. Hence, scavenging H_2_O_2_ by natural antioxidants sources is important for protection of biological systems.

#### 2.2.4. Reducing Power (RP) Assay

Reductive capabilities of plant extracts can serve as a significant indicator of their potential antioxidant activities [40]. The potassium ferricyanide reduction method is a widely used method for evaluating the RP of plant polyphenols. In this assay, the presence of antioxidants in test samples resulted in the reduction of the Fe^3+^/ferricyanide complex to the ferrous form by donating an electron. The Fe^2+^ was then monitored by measuring the formation of Perl’s Prussian blue [36]. Increasing absorbance of the reaction mixture at 700 nm indicates an increase in the RP. The reducing ability of grape cane extracts (50, 100, 150, 200 and 250 μg/mL) and standard antioxidants (25, 50, 75, 100 and 125 μg/mL) are presented in Figure 3D. All the extracts were capable of reducing Fe^3+^ and did so in a linear dose-dependent manner. Grape cane extract from the cultivar ‘Junzi’ exhibited the strongest RP (1.38 ± 0.06) which was significantly higher than Trolox (0.81 ± 0.05), while ‘Chardonnay’ yielded the weakest (0.74 ± 0.04) at a concentration of 100 μg/mL. Gallic acid and TBHQ showed rather prominent reductive capabilities at the same concentration, with the RP values of 2.67 ± 0.11 and 2.20 ± 0.07, respectively. As shown in Table 2, the rank order of EC_50_ values for RP was: Gallic acid > TBHQ > Junzi > Shuangyou ≥ Hongmeigui > Beibinghong > Baiyu ≥ Cabernet Sauvignon > Pinot Noir > Shuanghong > Trolox > Maoputao > Victoria Blanc > Chardonnay. A correlation test showed that the EC_50_ values of grape canes extracts were inversely correlated with their TPC (*r* = −0.804, *p* < 0.01) and TFC (*r* = −0.749, *p* < 0.01), supporting the former statement on the contribution of phenolic compounds in the antioxidant activities of grape cane extracts through the previous radical-scavenging models.

### 2.3. Identification and Determination of Phenolic Constituents in Extracts

In order to know the phenolic constituents present in the grape cane extract, all the extracts were analyzed by HPLC-DAD-UV. Chromatographic identification and confirmation of phenolic compounds were based on comparing retention times with authentic standards and UV absorption spectrum data. Gallic acid, protocatechuic acid, vanillic acid, syringic acid, (+)-catechin, (−)-epicatechin, and *trans*-resveratrol could be found in the extracts (Table 3). These phenolic compounds, occurring in lignified grapevine organs/tissues such as grape canes [12,15], phloem and xylem of one-year-old canes [14] and shoots [13], have been well identified in previous studies. The results of Duncan's new multiple range test analysis revealed that significant differences were observed between the grape cane extracts of different cultivars with respects to their phenolic compounds.

**Table 3:** Phenolic composition (mg/g extract)of the investigated grape cane extracts.

Species/cultivars	GA ^a^	PA	VA	SYA	CAT	EC	RES
***V. amurensis***							
Shuangyou	0.28 ± 0.01 c	1.19 ± 0.05 g	0.42 ± 0.02 f	0.93 ± 0.04 c	5.64 ± 0.22 b	5.98 ± 0.23 c	10.95 ± 0.43 b
Shuanghong	0.42 ± 0.02 f	0.89 ± 0.03 c	0.39 ± 0.02 de	0.84 ± 0.03 b	6.88 ± 0.27 d	7.02 ± 0.28 d	9.88 ± 0.39 b
Beibinghong	0.37 ± 0.01 e	0.98 ± 0.04 de	0.37 ± 0.01 cd	0.88 ± 0.03 bc	7.24 ± 0.28 de	7.55 ± 0.30 e	12.32 ± 0.48 c
***V. pentagona***							
Maoputao	0.41 ± 0.03 f	0.91 ± 0.04 c	0.40 ± 0.02 ef	0.58 ± 0.02 a	6.21 ± 0.24 c	6.03 ± 0.24 c	14.33 ± 0.56 ef
***V. davidii***							
Junzi	0.43 ± 0.03 f	1.32 ± 0.05 h	0.45 ± 0.02 g	1.07 ± 0.04 d	12.34 ± 0.48 g	11.22 ± 0.44 g	7.33 ± 0.28 a
Baiyu ^w^	0.44 ± 0.02 f	1.03 ± 0.04 ef	0.39 ± 0.02 de	0.89 ± 0.03 bc	9.32 ± 0.37 f	9.01 ± 0.35 f	6.58 ± 0.26 a
***V. vinifera***							
Cabernet Sauvignon	0.41 ± 0.01 f	0.93 ± 0.04 cd	0.42 ± 0.02 f	1.22 ± 0.05 e	6.32 ± 0.25 c	6.15 ± 0.24 c	13.58 ± 0.53 de
Hongmeigui	0.33 ± 0.03 d	0.68 ± 0.03 b	0.37 ± 0.01 cd	0.88 ± 0.03 bc	7.03 ± 0.28 de	6.87 ± 0.27 d	15.42 ± 0.60 f
Pinot Noir	0.35 ± 0.03 de	1.08 ± 0.04 f	0.35 ± 0.01 c	1.02 ± 0.04 d	7.52 ± 0.30 e	7.12 ± 0.28 de	18.99 ± 0.75 g
Chardonnay ^w^	0.17 ± 0.01 a	0.53 ± 0.02 a	0.29 ± 0.01 b	0.93 ± 0.04 c	4.29 ± 0.17 a	4.11 ± 0.17 a	12.64 ± 0.50 cd
Victoria Blanc ^w^	0.24 ± 0.01 b	0.69 ± 0.03 b	0.25 ± 0.01 a	0.88 ± 0.03 bc	5.31 ± 0.21 b	4.98 ± 0.20 b	13.33 ± 1.45 cd

^a^ Values (mean ± SD, *n* = 3; mg/g dry extract) with the same lowercases are not significantly different within each column according to Duncan's new multiple range test (*p* < 0.05). GA, gallic acid; PA, protocatechuic acid; VA, vanillic acid; SYA, syringic acid; CAT, (+)-catechin; EC, (−)-epicatechin; RES, *trans*-resveratrol. ^w^ White grape cultivars. Others are red ones.

In general, the phenolic profiles of grape cane extracts were dominated by three phenolic categories, phenolic acids, flavonoids and stilbene. The concentrations of phenolic acids were extremely low when compared with the other two phenolic groups. Of phenolic acids, the most abundant compound in the extracts was identified to be protocatechuic acid, ranging from 0.53 (Chardonnay) to 1.32 mg/g dry extract (Junzi), followed by syringic acid from 0.58 (Maoputao) to 1.22 mg/g dry extract (Cabernet Sauvignon). The amounts of gallic acid and vanillic acid in grape cane extracts, with mean values (*n* = 11) of 0.35 mg/g dry extract and 0.37 mg/g dry extract, respectively, were approximately three-folds lower than those of protocatechuic acid and syringic acid. For flavonoids, both (+)-catechin and (−)-epicatechin exhibited comparable levels in all grape cane extracts, with average values of 7.10 mg/g dry extract and 6.92 mg/g dry extract, respectively. The extracts from the cultivars ‘Junzi’ and ‘Baiyu’ had significantly higher contents of (+)-catechin and (−)-epicatechin than did other cultivars (*p* < 0.05). The highest *trans*-resveratrol content was detected in ‘Pinot Noir’ (14.33 mg/g dry extract), followed by ‘Cabernet Sauvignon’ and ‘Victoria Blanc’ and the lowest was found in ‘Baiyu’ (6.58 mg/g dry extract). These results indicated that quantitative distribution of phenolic compounds in grape cane extracts of the eleven cultivars examined was strongly influenced not only by their genetic background, but also by production sites and other factors. This is in agreement with the previous observations that the phenolic content in grape skin and seed vary depending on varietal and environmental factors [26,27]. Regarding the contents of phenolic compounds, comparison of results with literature data is difficult due to different authors expressing results in different ways. Zhang *et al*. investigated *trans*-resveratrol in one-year-old cane samples of 118 grape cultivars (mainly belonging to *V. vinifera*, *V. labrusca*, and *V. labrusca* and *V. vinifera* hybrids) and found the content of *trans*-resveratrol ranging from 320.6 to 1751.6 mg/kg fresh cane [12]. In another study, the concentration of *trans*-resveratrol in grape cane of ‘Pinot Noir’ (*V. vinifera*) was found to be 3.45 mg/g dry cane [41].

Most of the phenolic compounds herein reported have been shown to possess promising biological properties, especially for their strong antioxidant and antiradical activities *in vitro* and *in vivo* [6,20,17,25]. The difference of phenolic composition might explain the different antioxidant abilities of grape cane extracts observed above. Also, it can be speculated that phenolic compounds present in the extracts may exert their antioxidant capacity individually as well as synergistically.

## 3. Experimental

### 3.1. Plant Materials and Chemicals

Ideal 1-year-old cane samples of moderate vigor (approximately 0.8–1.0 cm diameter) were collected during the 2008 pruning period. Five *V. vinifera* grape canes of ‘Cabernet Sauvignon’, ‘Hongmeigui’, ‘Pinot Noir’, ‘Chardonnay’ and ‘Victoria Blanc’ were taken from the experimental vineyard of grape germplasm repository in the College of Enology, Northwest A&F University (Yangling, Shaanxi Province). Six Chinese wild grape canes including ‘Shuangyou’, ‘Shuanghong’ and ‘Beibinghong’ of *V. amurensis* (Tonghua, Jilin Province), ‘Maoputao’ of *V. pentagona* (Lantian, Shaanxi Province) and ‘Baiyu’ and ’Junzi’ of *V. davidii* (Chongyi, Jiangxi Province) were collected from their native habitats. All the chopped cane samples were frozen in liquid nitrogen, transported to our laboratory, freeze-dried (Model 50-SRC-5, VirTis, Gardiner, NY, USA), ground through a 0.5-mm sieve using a domestic electrical grinder (final particle size < 0.5 mm), stored in labeled plastic bags under vacuum and then stored at −20 °C in a freezer until extraction.

The reagents used in this study, such as 2,2-diphenyl-1-picrylhydrazyl (DPPH), 2,2-azinobis(3-ethyl-benzothiazoline-6-sulfonic acid) (ABTS), 2-(1,1-dimethylethyl)-1,4-benzenediol (TBHQ), 3,3'- (3,3'- dimethoxy- [1,1'- biphenyl]- 4,4'- diyl)bis[2- (4- nitrophenyl)- 5- phenyl- 2*H*- tetrazol- 3- ium] dichloride (NBT), 1,4-dihydronicotinamide adenine dinucleotide (NADH), 5-methylphenazinium methosulfate (PMS), 2-deoxyribose (DR), Folin-Ciocalteu’s phenol regent (FCR), 6-hydroxy-2,5,7,8-tetramethylchromane-2-carboxylic acid (Trolox), and all the phenolic compounds (purity >97%) were supplied by Sigma-Aldrich (Shanghai, China). HPLC grade methanol, acetonitrile and analytical grade acetic acid were purchased from Tianjin Kermel Chemical Reagent Co. Ltd. (Tianjin, China). Water was purified using the Milli-Q system (Millipore, Bedford, MA, USA). All other chemicals were analytical grade supplied by Xi’an Chemical Reagent Co. Ltd. (Xi’an, China).

### 3.2. Extraction

A fine dried grape cane powder sample (100 g) was extracted three times with acidified methanol solution (1000 mL, 1 N HCl/methanol/water, 1/80/19, v/v/v) in a shaking incubator for 24 h at 20 °C to avoid thermal degradation. The supernatant and the sediment were separated by vacuum-filtration. The solvent of the combined methanol extracts was evaporated in a Büchi RE-111 evaporator (Buchs, Switzerland) at 35 °C and the remaining water was removed by lyophilization to obtain methanolic extract. All extracts were re-dissolved in methanol and stored at −20 °C for further analysis.

### 3.3. Determination of Total Phenolic and Total Flavonoid Contents

Total phenolic content of each extract was determined by the Folin-Ciocalteu colorimetric method [42] using gallic acid as standard. In short, an aliquot of sample solution (0.1 mL, all of the solutions were diluted with methanol to adjust the absorbance values within the calibration range) was thoroughly mixed with FCR (0.3 mL) and distilled water (3.0 mL), and 20% Na_2_CO_3_ (1.0 mL) was added after 8 min. The mixture was kept in the dark at room temperature for 2 h. Absorbance of samples was measured at 765 nm (UV-1700, Shimadzu Corp, Kyoto, Japan) against a blank (methanol) similarly prepared. The total phenolic contents of grape cane extracts were determined through the calibration curve (*y* = 0.0022*x* + 0.0013, *R^2^* = 0.9997; *y* and *x* are the values of the absorbance and solution concentration, respectively), which was performed with a series of gallic acid solutions (40–400 mg/L). The results were expressed as the equivalent to milligrams of gallic acid per gram of dry extract (mg GAE/g). 

Total flavonoid content of each extract was determined through the aluminum chloride (AlCl_3_) colorimetric method [43] using quercetin as standard. Briefly, an aliquot of sample solution (0.5 mL) was mixed with 10% AlCl3 (0.1 mL), 1 M potassium acetate (0.1 mL) and distilled water (2.5 mL). The mixture remained at room temperature for 30 min. Absorbance of samples was measured at 415 nm versus a blank. The TFC was determined through the calibration curve (*y* = 0.0026*x* + 0.0009, *R^2^* = 0.9993); *y* and *x* are the values of the absorbance and solution concentration, respectively), which was carried out with a series of quercetin solutions (12–120 mg/L). The results were expressed as the equivalent to milligrams of quercetin per gram of dry extract (mg QCE/g). 

### 3.4. Determination of Free Radicals and Hydrogen Peroxide Scavenging Activities

The DPPH assay was done according to the previous procedure [44] with minor modifications. An aliquot of sample (0.1 mL) at various concentrations was added to 63 μM freshly prepared DPPH radical (DPPH^•^) methanol solution (3.9 mL). An equal volume of methanol and DPPH^•^ served as a control. The reaction mixtures were shaken vigorously and incubated at 37 °C in the dark for 1 h. The absorbance (Abs) was recorded at 517 nm.

The ABTS assay was carried out according to the previous method [45] with minor modifications. ABTS radical cation (ABTS^•+^) was generated by reacting ABTS solution (7 mM) with 2.45 mM potassium persulfate and allowing the mixture to stand in the dark at room temperature for 16 h. The ABTS^•+^ solution was diluted with methanol to give an absorbance at 734 nm of 0.70 ± 0.02 in a 1-cm cuvette. After addition of diluted ABTS^•+^ solution (2.0 mL) to samples (20 μL) at various concentrations the Abs reading was taken at 30 °C exactly 6 min after initial mixing. An equal volume of methanol and ABTS^•+^ served as a control.

Measurement of superoxide radical (O_2_^•−^) scavenging activities of samples were based on the method of Nishikimi *et al*. [34] with slight modifications. The reaction mixture, which contained samples (0.1 mL, 0–2 mg/mL) in methanol, NBT (1 mL, 156 μM) in phosphate buffer (100 μM, pH 7.4), NADH (1 mL, 468 μM) in phosphate buffer, and PMS (0.1 mL, 60 μM), was incubated at room temperature for 5 min, and its Abs was read at 560 nm against methanol as control.

The scavenging capacity of samples for hydroxyl radical (OH^•^) was estimated according to a modified method [39]. An aliquot of samples (0.1 mL, 0–10 mg/mL) was incubated with 3.75 mM DR (0.5 mL), 60 mM potassium phosphate buffer (pH 7.4, 2.9 mL), 5 mM FeSO_4_-EDTA (0.5 mL), and 1 mM H_2_O_2_ (0.5 mL) for 60 min at 37 °C. Then the reaction mixture (1 mL) was added to thiobarbituric acid (1 mL, 1%, w/v) and trichloroacetic acid (TCA, 1 mL, 2%, w/v); the tubes were heated in a boiling water bath for 15 min to develop the pink chromogen measured at 532 nm. The reaction mixture without samples was used as control.

The hydrogen peroxide (H_2_O_2_) scavenging capacities of samples were estimated by the previous method [46] with minor modifications. The reaction mixture contained samples (0.5 mL, 0–0.4 mg/mL) and 10 mM H_2_O_2_ (2.5 mL) in phosphate buffer (pH 7.4), and the Abs was recorded at 230 nm after 10-min incubation against reagent blank solution. The reaction mixture without samples was control.

The scavenging activities were estimated based on the percentage of DPPH^•^/ABTS^•+^/O_2_^•−^/OH^•^/H_2_O_2_ scavenged as the following equation:

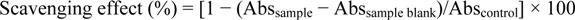


EC_50_ values, which are the concentration of sample required for 50% scavenging of DPPH^•^/ABTS^•+^/ O_2_^•−^/OH^•^/H_2_O_2_ in the specified reaction time, were calculated from the graph plotting scavenging percentage against sample concentration. Gallic acid, Trolox, and TBHQ were used as positive controls in these assays.

### 3.5. Determination of Reducing Power

The reducing power of samples was measured according to the previous method [36] with a slight modification. An aliquot of samples (1 mL), with different concentrations, was mixed with 200 mM phosphate buffer (2.5 mL, pH 6.6) followed by of 1% potassium ferricyanide [K_3_Fe(CN)6, 2.5 mL]. The mixture was incubated for 20 min in a water bath at 50 °C. After incubation, 10% TCA (1 mL) was added, followed by centrifugation at 3000 × *g* for 10 min. The supernatant (2.5 mL) was mixed with distilled water (2.5 mL) and 0.1% ferric chloride (0.5 mL). Then the Abs was measured at 700 nm against a blank. EC_50_ value (μg extract/mL) is the effective concentration at which the Abs is 0.5 for reducing power and was obtained by interpolation from the linear regression analysis. Gallic acid, Trolox, and TBHQ were used as positive controls in these assays.

### 3.6. HPLC Analysis of Grape Cane Extracts

Grape cane extracts were analyzed using a Shimadzu liquid chromatograph unit (LC-2010AHT, Kyoto, Japan) comprising a quaternary pump, a photodiode array detector (DAD), a UV-Vis detector, a Shim-Pack VP-ODS C_18_ column (250 mm × 4.6 mm, 5 μm) and an autosampler. Each lyophilized extract (20 mg) was dissolved in HPLC grade methanol (10 mL). The standards, gallic acid (GA), protocatechuic acid (PA), (+)-catechin (CAT), vanillic acid (VA), syringic acid (SYA), (−)-epicatechin (EC), and *trans*-resveratrol (RES), were dissolved in methanol at a stock concentration of 1 mg/mL. Calibration standard mixture was prepared by appropriate dilutions with methanol from the stock solution. All solutions were stored in the dark at −40 °C and filtered through 0.22-µm membranes prior to injection.

A gradient solvent system was employed with solvent A being water-acetic acid (97:3, v/v) and solvent B being acetonitrile. The elution profile had the following proportions (v/v) of solvent B: 0.00–5.00 min, 0–8.5%; 5.00–16.50 min, 8.5–2.0%; 16.50–35.00 min, 2.0–18%; 35.00–50.00 min, 18–20%; 50.00–65.00 min, 20–30%; 65.00–70.00 min, 30–0%. The wavelength-switching program was employed. The column was held at 30 °C and was flushed at a flow rate of 0.8 mL/min. A volume of 10 µL was injected for each run in triplicate. The DAD detector was applied to scan (200–400 nm) the phenolic compounds of interest to ascertain their maximum absorbance wavelengths and acquire other spectral information. The UV detector was used for quantitative purposes with the external standard. The linearity of the method was established by automatic injections of the standard mixture solutions at six calibration levels from low to high concentrations. Results were acquired and processed by the Shimadzu Workstation CLASS-VP 6.12 software.

### 3.7. Statistical Analysis

All determinations were done at least in triplicate and were expressed as means ± standard deviations (SD). Statistical analyses were performed using SPSS (SPSS, Inc., Chicago, IL, USA) version 10.0 for Windows. Simple regression analysis was used to calculate the concentration-response relationship of standard solution for calibration as well as samples. Duncan’s multiple range test and Pearson’s correlation coefficients (*r*) were conducted to compare the data.

## 4. Conclusions

The results of this study clearly indicate that methanolic extracts from grape canes contain a considerable amount of phenolics and possess significant *in vitro* antioxidant and antiradical capacities, although the order of antioxidant potency of each cultivar evaluated by different models does not follow the same pattern. In general, strong and positive associations were observed between antioxidant activities and total phenolic content as well as total flavonoids content. With special attention to scavenging effects against different ROS, most of the extracts were found to be more efficient than gallic acid, Trolox and TBHQ. Hence, grape cane extracts should be treated as potential free radical scavengers. Qualitative and quantitative analysis of phytocompounds in methanolic extracts by HPLC-DAD-UV suggested that (+)-catechin, (−)-epicatechin, and *trans*-resveratrol were present at the highest concentrations in all extracts. Grape canes, as neglected agricultural pruning wastes from grape and wine industry, are good candidates for further development as nutraceutical supplements or antioxidant remedies. Future studies should focus on the assessments of economic benefits and *in vivo* activities of these extracts before their commercial exploitation.
